# Therapeutic effect and safety of individualized chemotherapy combined with sequential immunotherapy based on BRCA1 mRNA expression level in unresectable pancreatic cancer

**DOI:** 10.3389/fonc.2022.1015232

**Published:** 2022-11-01

**Authors:** Juan Du, Linxi Zhu, Huizi Sha, Zhengyun Zou, Jie Shen, Weiwei Kong, Lianjun Zhao, Qing Gu, Lixia Yu, Yudong Qiu, Baorui Liu

**Affiliations:** ^1^ The Comprehensive Cancer Center of Drum Tower Hospital, Medical School of Nanjing University, Nanjing, China; ^2^ Department of Hepatopancreatobiliary Surgery, Drum Tower Hospital, Medical School of Nanjing University, Nanjing, China; ^3^ National Institute of Healthcare Data Science, Nanjing University, Nanjing, China

**Keywords:** Pancreatic cancer, BRCA1 mRNA, Individualized chemotherapy, Immunotherapy, Adverse effect

## Abstract

**Aim:**

We aimed to evaluate the efficacy and safety of individualized chemotherapy combined with sequential immunotherapy based on BRCA1 mRNA expression in unresectable pancreatic cancer.

**Methods:**

The expression of BRCA1 mRNA in tumor tissues of 25 patients with pancreatic cancer was detected in this retrospective study. Patients in the medium and high expression groups were treated with paclitaxel-based chemotherapy: albumin paclitaxel 125mg/m^2^, gemcitabine 1g/m^2^, day 1. Patients in the low expression group were treated with oxaliplatin-based chemotherapy: oxaliplatin 85mg/m^2^, gemcitabine 1g/m^2^, day 1. Sequential GM-CSF and IL-2 immunotherapy were applied. Patient condition, treatment efficacy and safety were assessed every 4 cycles.

**Results:**

A total of 25 patients were enrolled in the study. All of them were observed for toxic side effects and 24 of them were evaluated for efficacy. The median overall survival and median progression-free survival were 11.9 months and 6.3 months. The disease control rate was 91.7%, of which 37.5% (9/24) patients achieved partial remission (PR), 54.2% (13/24) patients achieved stable disease (SD) and 8.3% (2/24) patients were assessed as progressive disease(PD). Of the 15 patients with medium or high expression in BRCA1 mRNA, 7 achieved PR and 8 achieved SD. Of the 9 patients with low BRCA1 mRNA expression, 2 achieved PR, 5 achieved SD and 2 had PD. The proportion of eosinophils in the blood of some patients with good therapeutic effects was significantly higher than that before treatment. Hematological and non-hematological toxicity during the treatment were mostly grade 1~2. The two most common grade 3 to 4 adverse events were fever and thrombocytopenia.

**Conclusion:**

Our results suggest that individualized selection of chemotherapy combined with sequential immunotherapy according to BRCA1 mRNA expression level in unresectable pancreatic cancer could control the disease and have controllable adverse reactions.

## Introduction

Pancreatic cancer is a kind of digestive tumor with relatively low incidence but high degree of malignancy. It is characterized by difficulty in early detection and invasive metastasis. Almost 85% patients are not suitable for radical surgery at the time of diagnosis. Together with high recurrence and metastasis rate after resection, the prognosis of pancreatic cancer is extremely poor. Although the surgical technique progresses rapidly and the postoperative mortality rate gradually decreases, the mortality rate of pancreatic cancer remains high (1). At present, the therapeutic effects of surgery, radiotherapy and chemotherapy for pancreatic cancer are not satisfactory and the median survival time is usually no more than 6 months (2). Pancreatic cancer is currently ranked 3rd in the United States for the cause of malignant tumor death (3) and ranked 9th in China (4). It is expected that pancreatic cancer will become the second leading cause of cancer death in the world in 2030 (5). After nearly one decade of clinical research, chemotherapy for advanced pancreatic cancer has formed a standard treatment regimen based on gemcitabine. Recent studies have shown that the nab-paclitaxel combined with gemcitabine chemotherapy regimen and the FOLFIRINOX regimen are significantly superior to gemcitabine monotherapy in the treatment of pancreatic cancer. However, these regimens also significantly increase toxic reactions. Most patients can not adhere to chemotherapy because of the toxic side effects such as myelosuppression and gastrointestinal reactions (6). Therefore, there is an urgent need to find new treatments or therapies to improve the prognosis of patients.

Although lots of cancers already have several biomarkers that could contribute to treatment decision making, there are currently no proven biomarker-based treatment options for pancreatic cancer. Previous studies have shown that BRCA1 is a bidirectional drug-sensitivity molecule with its expression level related to the sensitivity to platinum and taxanes (7). Tumors with high BRCA1 expression levels are sensitive to paclitaxel, while tumors with low BRCA1 expression are sensitive to platinum. Individualized selection of chemotherapy drugs based on BRCA1 mRNA expression level may improve the efficacy of chemotherapy and prolong the survival time of patients. Tumor immunotherapy is a new focus of anti-tumor therapy after surgery, radiotherapy, chemotherapy and targeted drugs. The combination of chemotherapy and immunotherapy could foster a synergistic effect and further improve the therapeutic properties.

To explore the role of BRCA1 mRNA expression in the chemotherapy of unresectable pancreatic cancer and the synergistic effect of chemotherapy and immunotherapy, we have carried out a retrospective study which focused on individualized chemotherapy combined with sequential immunotherapy according to BRCA1 mRNA expression in the first-line treatment of unresectable pancreatic cancer. We evaluated the effect of these regimens on objective response rates, disease control rates, survival conditions and safety in unresectable pancreatic cancer.

## Materials and methods

### Patients

25 patients with unresectable pancreatic cancer who were diagnosed by surgery or EUS-FNA biopsy from November 2017 to November 2018 in Nanjing Drum Tower Hospital were included in this retrospective study (8). Among them, 10 cases (40%) had locally advanced pancreatic cancer, 14 cases (56%) had metastatic pancreatic cancer, and 1 case (4%) had recurrence after radical resection of pancreatic cancer. 11 of them (44%) were male and 14 (56%) were female. The patients ranged from 48 to 72 years old with a median age of 66.5 years. 15 cases were pancreatic head or neck tumors. 10 cases were pancreatic body or tail tumors. All patients signed a written informed consent form prior to the treatment.

### Detection of BRCA1 mRNA expression level

RNA was extracted from paraffin sections of patient tumor specimens. The RNA was reverse transcribed into cDNA with MMV-L reverse transcriptase (product of Takara) according to the protocol. Real-time quantitative PCR was performed on a Stratagene MX3000P type real-time PCR instrument. The forward primer sequence was GAAACCGTGCCAAAAGACTTC and the reverse primer sequence was CCAAGGTTAGAGAGTTGGACAC. Three replicate wells were made for each gene. Quantification of gene expression was performed using the ABI Prism 7900HT Sequence Detection System (Applied Biosystems). Relative gene expression quantifications were calculated according to the comparative Ct method using β-actin as an endogenous control and commercial human lung and liver RNA (Stratagene, La Jolla, CA, USA) as calibrators. Final results were determined by the formula 2^-(ΔCtsample-ΔCtcalibrator)^ and were performed according to Technical Bulletin #2 (Applied Biosystems) (9).

### Study design and treatment regimens

According to BRCA1 detection databases in Nanjing Drum Tower Hospital Cancer Laboratory, there were more than 1000 specimens detected over the last 10 years. We took the upper 1/3 as high expression, the lower 1/3 as low expression and the middle 1/3 as medium expression (7, 10–13). Patients were divided into low, medium and high expression groups according to their BRCA1 mRNA expression level. Patients in the medium and high expression groups were treated with paclitaxel-based chemotherapy: albumin paclitaxel 125mg/m^2^, gemcitabine 1g/m^2^, day 1. Patients in the low expression group were treated with oxaliplatin-based chemotherapy: oxaliplatin 85mg/m^2^, gemcitabine 1g/m^2^, day 1. Both groups received the follow-up immunotherapy. From day 3 to 7, the patients underwent subcutaneous injection of granulocyte mononuclear colony-stimulating factor (GM-CSF), 150 μg, QD. From day 8 to 14, the patients underwent subcutaneous injection of recombinant human interleukin-2 (IL-2), 100 WU, BID. 14 days was regarded as a cycle. Chest and abdominal CT were performed every 4 cycles to assess objective efficacy and whether the disease progressed. If the disease didn’t progress, the patient would maintain treatment after 12 cycles of the original regimen. If the disease progressed, the original regimen was replaced with a second-line treatment regimen. Before each cycle of chemotherapy, serum tumor markers were detected to observe their dynamic changes. The follow-up method was a combination of inpatient, outpatient and telephone follow-up.

### Study endpoints and efficacy evaluation

The primary efficacy end point of the study was overall survival (OS), which was defined as the time from patient enrollment to the death (died from the primary tumor or complication) or the last follow-up (loss of follow-up) of the patient. The secondary efficacy end point was progression-free survival (PFS), which was defined as the time from patient enrollment to imaging confirmation of disease progression. Objective efficacy was assessed according to RECIST efficacy evaluation criteria and was divided into complete response (CR), partial response (PR), disease stabilization (SD) and progressive disease (PD). The objective response rate was defined as the sum of CR and PR. The disease control rate was defined as the sum of CR, PR and SD. Adverse reaction assessment was performed according to NCI-CTCAE v5.0 standard.

### Statistical analysis

All results were statistically analyzed using SPSS 24.0 statistical software. Independent sample t test was used to assess age differences between groups. Chi-square test was used to assess gender differences between groups. Kruskal-Wallis test was used to analyze the relationship between BRCA1 mRNA expression and chemotherapy efficacy. Survival analysis was performed by Kaplan-Meier method and differences between groups were tested by log-rank test. All tests were two-tailed with the test level a = 0.05.

## Results

### Patient characteristics

A total of 25 patients were enrolled in the study. The mean age of the patients was 66.5 years (48-72), including 11 males (44%) and 14 females (56%). There were 10 cases (40%) of locally advanced pancreatic cancer, 14 cases (56%) of metastatic pancreatic cancer and 1 case (4%) of postoperative recurrence of pancreatic cancer. According to the results of BRCA1 mRNA detection, 12 patients had high expression of BRCA1 mRNA (48%), 4 patients had medium expression of BRCA1 mRNA (16%), 8 patients had low expression of BRCA1 mRNA (32%) and 1 patient had no detectable BRCA1 mRNA expression (4%). The medium and high expression patients were assigned to the nab-paclitaxel group. The low and non-expression patients were assigned to the oxaliplatin group. There were no statistical differences in age (P=0.715) and diagnosis (P=0.284) between groups. There was a significant difference in gender (P=0.002) between groups. The baseline characteristics of the patients are shown in **Table 1**.

**Table 1:** Baseline characteristics of the 25 patients with unresectable pancreatic cancer.

Characteristics		GEM+nab-paclitaxel (N=16)	GEM+oxaliplatin (N=9)	*P*
**Age**	**Mean**	61.19 ± 7.45	60.11 ± 6.01	0.715
**Gender**	**Female**	13 (81.3%)	1 (11.1%)	0.002
	**Male**	3 (18.8%)	8 (88.9%)	
**Diagnosis**	**Metastasis**	7 (43.8%)	7 (77.8%)	0.284
	**Locally advanced**	8 (50.0%)	2 (22.2%)	
	**Recurrence**	1 (6.3%)	0 (0.0%)	

### Objective chemotherapy efficacy evaluation

Of the 25 patients with unresectable pancreatic cancer, 24 had objective efficacy evaluation. One patient did not complete the four-cycle treatment, so objective efficacy evaluation was not performed. Of the 24 patients, 9 had partial remission (37.5%), 13 had stable disease (54.2%) and 2 had progressive disease (8.3%). The overall objective response rate and disease control rate were 37.5% and 91.7% respectively. The objective response rate of the nab-paclitaxel group was 46.7% and the disease control rate was 100%. The objective response rate of the oxaliplatin group was 22.2% and the disease control rate was 77.8%. Independent sample Kruskal-Wallis test showed no significant difference in treatment efficacy between the two groups (P = 0.209). Further information is shown in **Table 2**.

**Table 2:** Efficacy of chemotherapy in the nab-paclitaxel group and oxaliplatin group.

Efficacy	Total (N=24)	GEM+nab-paclitaxel (N=15)	GEM+oxaliplatin (N=9)	*P*
**Partial Remission**	9 (37.5%)	7 (46.7%)	2 (22.2%)	0.209
**Stable Disease**	13 (54.2%)	8 (53.3%)	5 (55.6%)	
**Progressive Disease**	2 (8.3%)	0 (0.0%)	2 (22.2%)	

### Survival analysis

**Figure 1:**
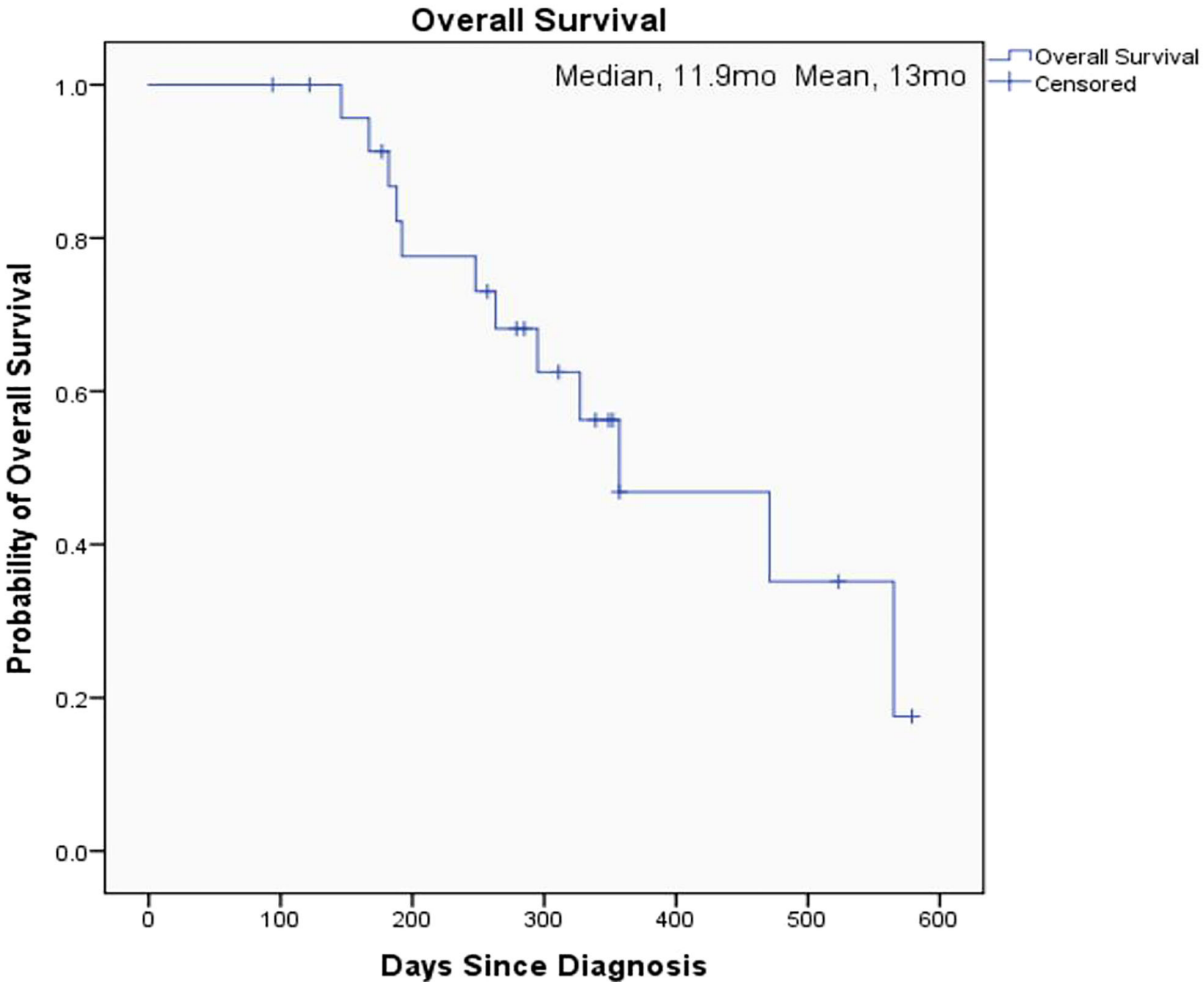
Kaplan-Meier Estimates of Overall Survival (all patients).

**Figure 2:**
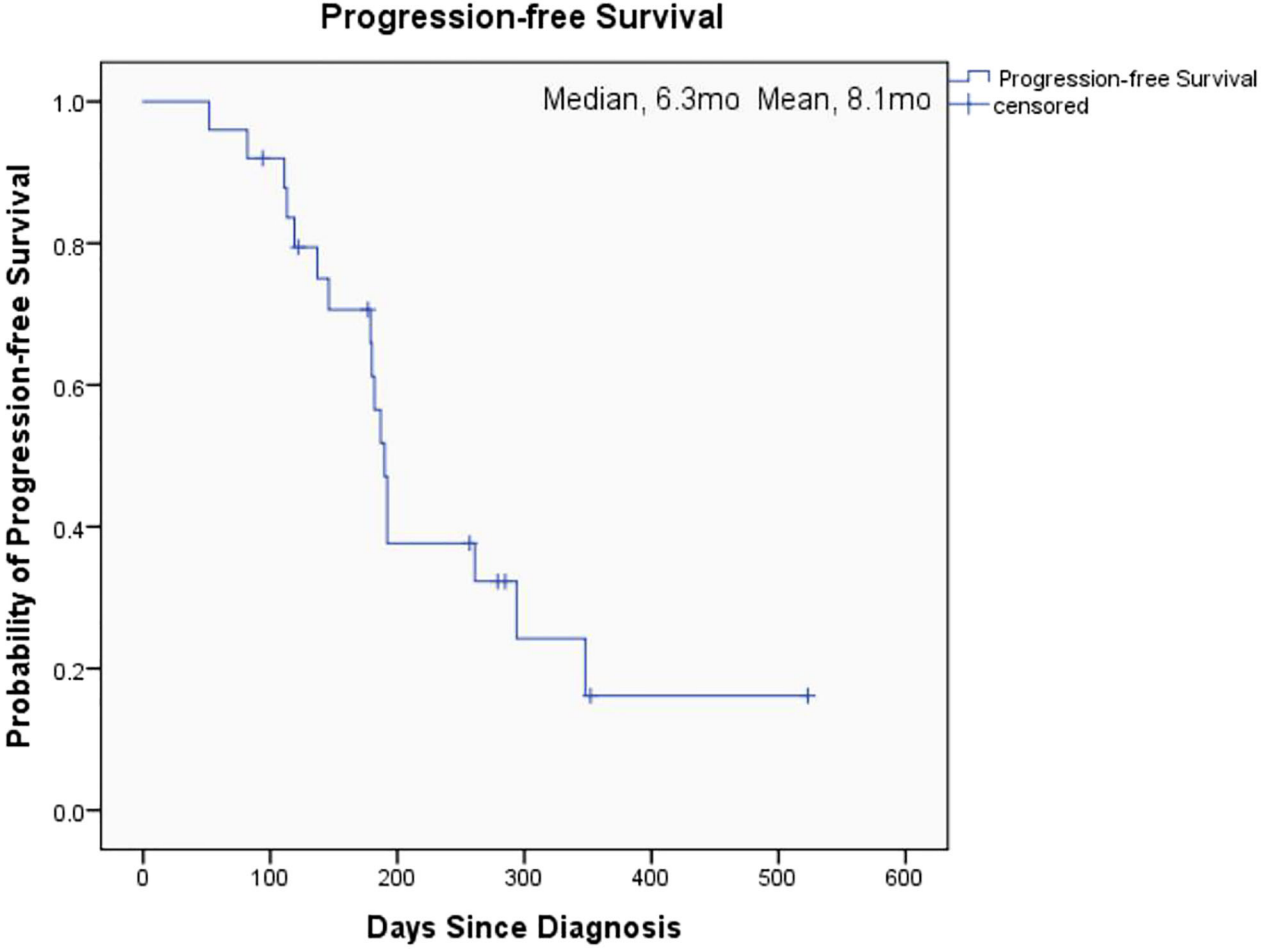
Kaplan-Meier Estimates of Progression-free Survival (all patients).

**Figure 3:**
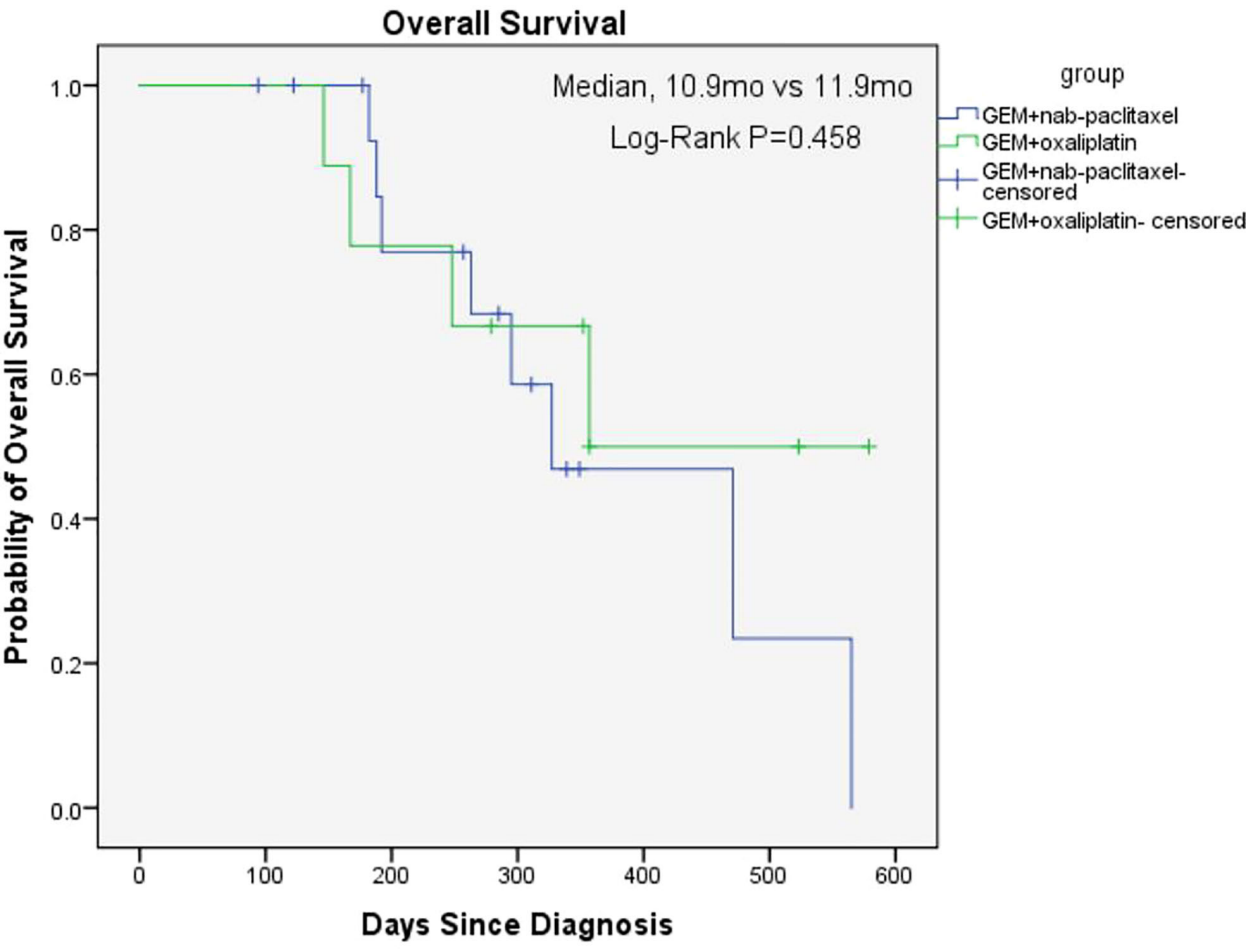
Kaplan-Meier Estimates of Overall Survival between two groups.

**Figure 4:**
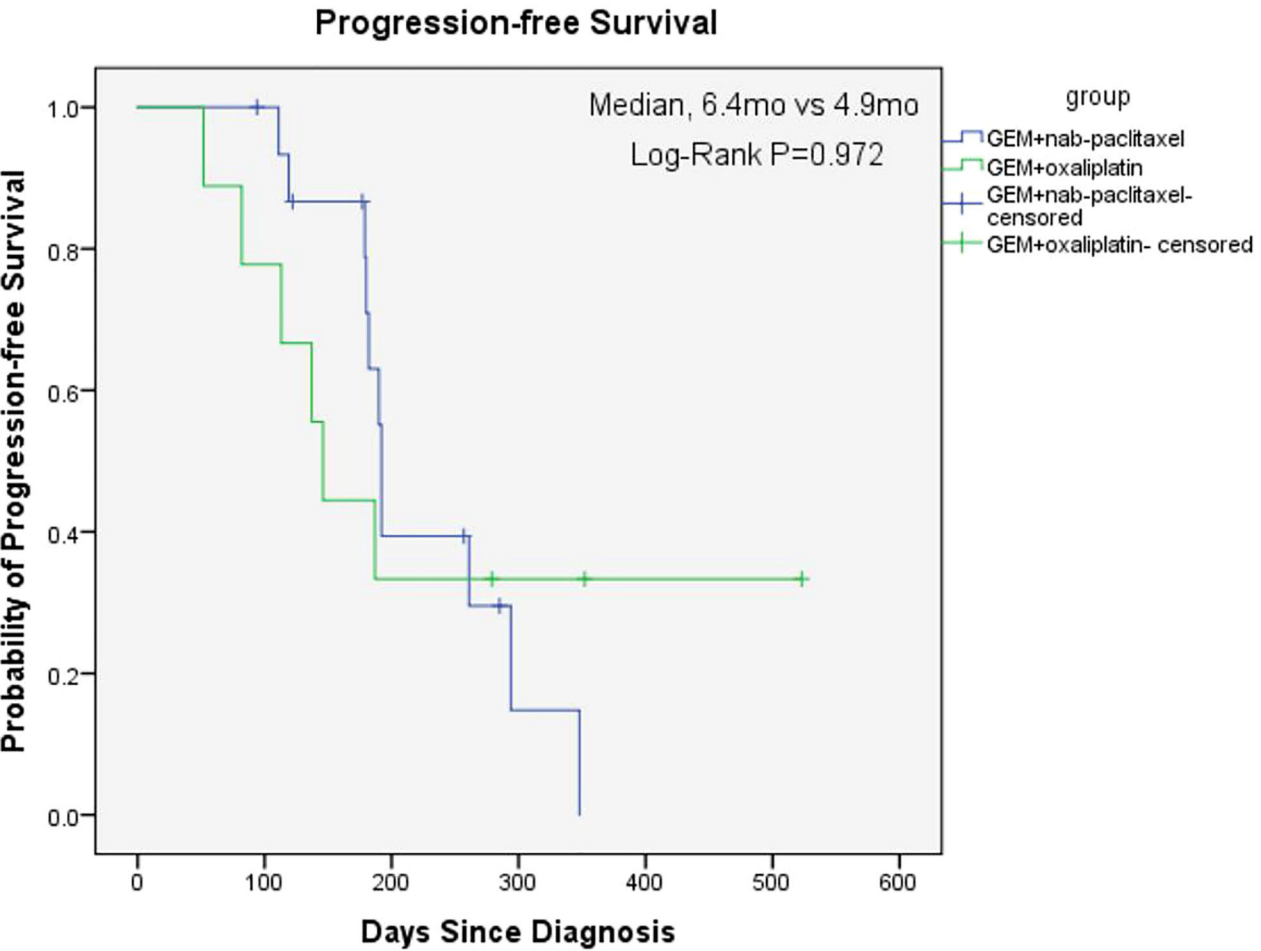
Kaplan-Meier Estimates of Progression-free Survival between two groups.

The overall median OS of the 25 patients with unresectable pancreatic cancer was 11.9 months (95% confidence interval [CI], 6.2 to 17.6) and the mean OS was 13 months (95% CI, 10.5 to 15.5) (**Figure 1**). The median PFS of the 25 patients was 6.3 months (95% CI, 5.9 to 6.7) and the mean PFS was 8.1 months (95% CI, 6.0 to 10.2) (**Figure 2**). The median OS of the 16 patients in the paclitaxel group was 10.9 months (95% CI, 5.8 to 16) and the median OS of the 9 patients in the oxaliplatin group was 11.9 months. There was no significant difference between the two groups (P=0.458) (**Figure 3**). The median PFS of the 16 patients in the paclitaxel group was 6.4 months (95% CI, 6.2 to 6.5) and the median PFS of the 9 patients in the oxaliplatin group was 4.9 months(95% CI, 3.9 to 5.7). There was no significant difference between the two groups (P=0.972) (**Figure 4**).

### Serum tumor marker CA19-9 during chemotherapy

**Figure 5:**
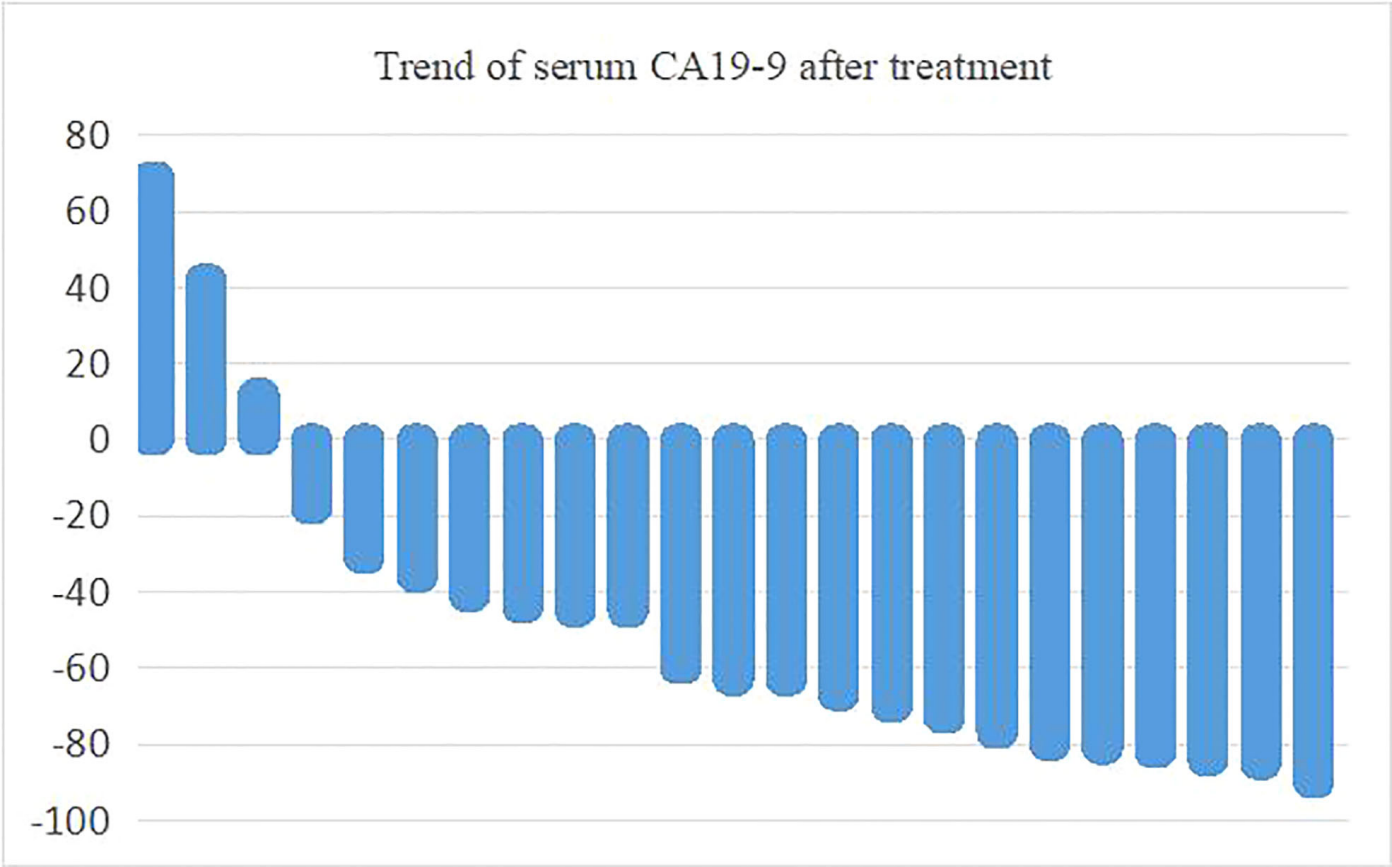
Trend of serum CA19-9 after treatment.

Of the 24 patients with efficacy evaluation, 23 had elevated serum carbohydrate antigen CA19-9 which decreased in most patients after treatment. Serum CA19-9 decreased by more than 50% in 13 patients. The maximum proportion of serum CA19-9 changes before and after treatment is shown in **Figure 5**.

### Changes of eosinophil count in peripheral blood

**Figure 6:**
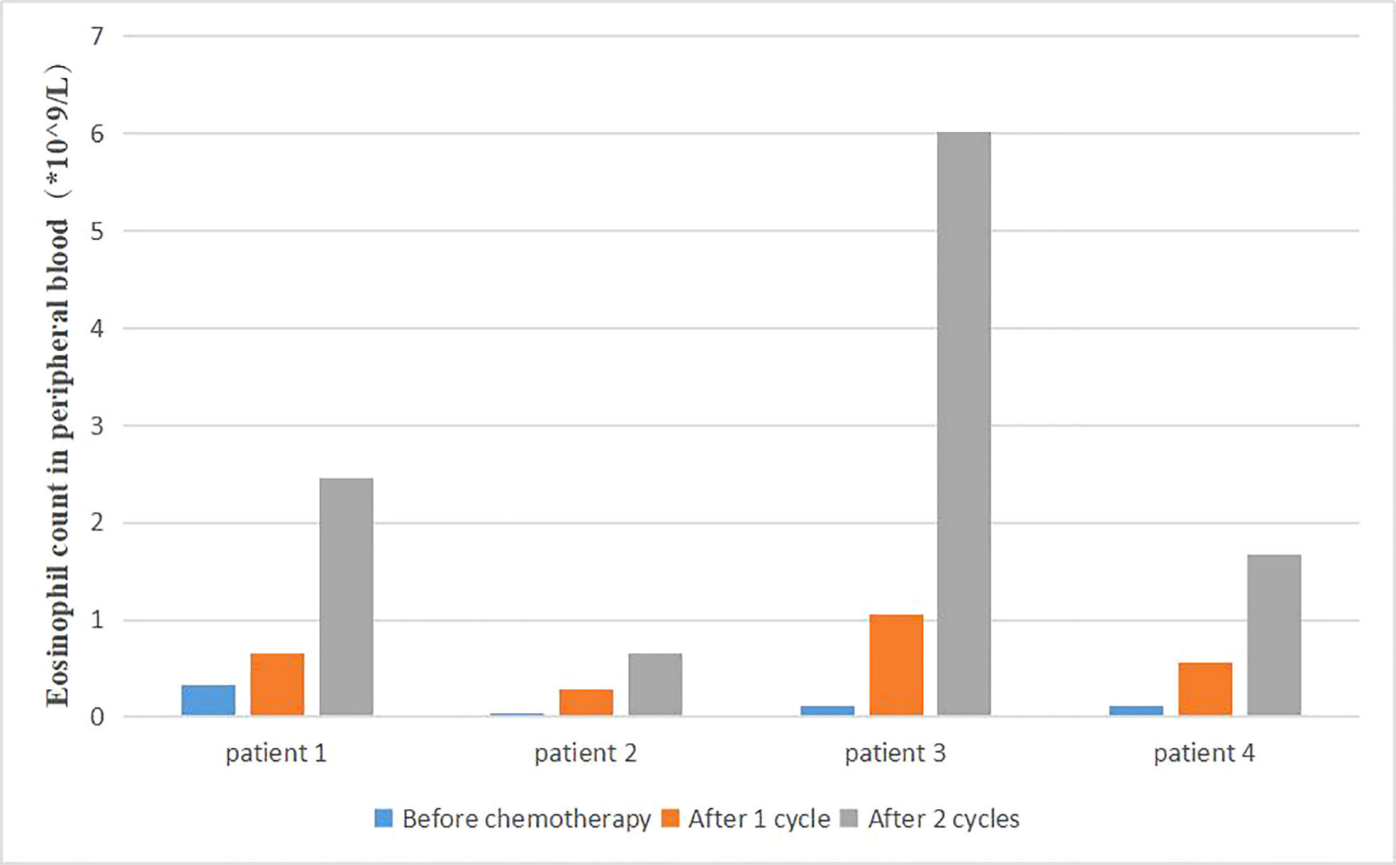
Changes of eosinophil count in peripheral blood during treatment.

Changes of patient blood routine were monitored during the treatment. We found that the proportion and count of eosinophils in some patients were significantly higher than before (**Figure 6**). The survival time of the same part of patients was also significantly prolonged. Due to the small number of patients, no statistical analysis was performed.

### Adverse reactions

The main adverse reactions during the treatment of the 25 patients are shown in **Table 3**. Common adverse reactions were fever, bone marrow suppression, gastrointestinal reactions, peripheral neurotoxicity and hair loss, most of which were 1 to 2 degrees. The most common grade 3 to 4 adverse events included leukopenia, neutropenia and thrombocytopenia, the incidence of which was 12%, 4% and 8%, respectively.

**Table 3:** Adverse reactions during treatment of the 25 patients.

Adverse reactions	Grade 1	Grade 2	Grade 1 + 2	Grade 3	Grade 4	Grade 3 + 4
**Leukopenia**	10 (40%)	3 (12%)	13 (52%)	2 (8%)	1 (4%)	3 (12%)
**Neutropenia**	7 (28%)	2 (8%)	9 (36%)	1 (4%)	0 (0%)	1 (4%)
**Thrombocytopenia**	5 (20%)	2 (8%)	7 (28%)	1 (4%)	1 (4%)	2 (8%)
**Anemia**	12 (48%)	2 (8%)	14 (56%)	0 (0%)	0 (0%)	0 (0%)
**Nausea**	6 (24%)	3 (12%)	9 (36%)	0 (0%)	0 (0%)	0 (0%)
**Vomiting**	4 (16%)	1 (4%)	5 (20%)	0 (0%)	0 (0%)	0 (0%)
**Fever**	12 (48%)	2 (8%)	14 (56%)	0 (0%)	0 (0%)	0 (0%)
**Muscle or joint pain**	6 (24%)	1 (4%)	7 (28%)	0 (0%)	0 (0%)	0 (0%)
**Hepatic dysfunction**	5 (20%)	1 (4%)	6 (24%)	0 (0%)	0 (0%)	0 (0%)
**Peripheral sensory neurotoxicity**	12 (48%)	3 (12%)	15 (60%)	0 (0%)	0 (0%)	0 (0%)
**Rash**	6 (24%)	2 (8%)	8 (32%)	0 (0%)	0 (0%)	0 (0%)
**Hair loss**	5 (20%)	19 (76%)	24 (96%)	0 (0%)	0 (0%)	0 (0%)

## Discussion

The aim of our study was to investigate the effect of individualized chemotherapy combined with sequential immunotherapy based on BRCA1 mRNA expression on the objective efficacy and survival conditions of patients with unresectable pancreatic cancer. Our study showed that individualized chemotherapy combined with sequential immunotherapy based on BRCA1 mRNA expression could control the disease well and increase patient survival time. In this single-center, retrospective study, although the 25 unresectable pancreatic cancer patients we included were mostly with advanced disease or distant metastasis, the objective response rate was 37.5% and the disease control rate was 91.7%. At the efficacy end point of our study, the median OS of all patients reached 11.9 months, with a median PFS of 6.3 months. This demonstrated that the new individualized chemotherapy combined with sequential immunotherapy could benefit patients with advanced pancreatic cancer and hence had important clinical value.

Chemotherapy is the primary treatment for unresectable pancreatic cancer. But for patients with locally advanced or metastatic pancreatic cancer, progression-free survival after chemotherapy is usually less than 3 months (14). In the past few years, two combined chemotherapy regimens have become new standards for first-line treatment of metastatic pancreatic cancer—FOLFIRINOX and AG (nab-paclitaxel combined with gemcitabine) regimen. The international MPACT study have shown that nab-paclitaxel combined with gemcitabine had good tolerance and significantly improved overall survival, progression-free survival and response rate compared with gemcitabine monotherapy. The median OS of the AG group was 8.7 months, with a median PFS of 5.5 months (15). The adverse effects of AG regimen appeared to be more controllable than FOLFIRINOX regimen in the ACCORD-11 trial (16), which meant it could be available to a wider range of patients. Therefore, AG regimen has become the first-line treatment for unresectable pancreatic cancer. However, these regimens have a high incidence of adverse reactions, such as bone marrow suppression, gastrointestinal reactions and other toxic and side effects, which can’t be tolerated by most patients in clinical application.

It has been confirmed in various models that defects in the function of BRCA gene increase the sensitivity of tumor cells to platinum drugs (17–22). Although BRCA mutation-associated pancreatic cancer is rare, the unique biological properties of this subgroup may benefit patients from treatment regimens targeting DNA damage repair pathways (23). In a retrospective study of BRCA-deficient pancreatic ductal adenocarcinoma (PDAC), results suggested that BRCA mutation status was a good prognostic indicator for early disease. Patients with BRCA mutation-related PDAC may benefit from treatment based on platinum drugs (24, 25). Therefore, the 2019 NCCN guidelines for pancreatic cancer indicated that if the patient had a BRCA1/2 gene mutation, it was recommended to choose a platinum-containing regimen. Similarly, the expression level of BRCA1 mRNA also could predict the chemotherapy efficacy of platinum drugs. Gao et al.’s study confirmed that patients with low BRCA1 mRNA expression were more likely to benefit from chemotherapy with platinum-containing regimens (7). Therefore, in this study, we gave individualized chemotherapy regimens to patients based on BRCA1 mRNA expression levels. The median OS and PFS of the patients were significantly longer than those of the MPACT trail, demonstrating that the efficacy of individualized chemotherapy regimen was superior to that of AG regimen regardless of the BRCA1 gene expression level. Currently, there is no clear and instructive biomarker for the selection of chemotherapy regimens for pancreatic cancer. Results of this retrospective study suggested that BRCA1 gene expression level may become a new biomarker for guiding chemotherapy options in patients with unresectable pancreatic cancer. Our study is expected to advance the field of biomarker research in individualized treatment of pancreatic cancer.

The synergistic application of chemotherapy and immunotherapy is a combination treatment that has been highly recommended in recent years. When chemotherapy and immunotherapy are used sequentially, chemotherapy could induce tumor cell apoptosis to release tumor-associated antigen, as well as improving tumor microenvironment, inhibiting Tregs and MDSCs, stimulating dendritic cells and antigen-specific cytotoxic T lymphocytes (CTL) to elicit a more effective immune response. The GOLFIG-2 trial was a clinical trail conducted by Correale et al. in advanced colon cancer.

Chemotherapy drugs commonly used in intestinal cancer, such as platinum oxalate, calcium leucovorin, fluorouracil combined with gemcitabine and GM-CSF, IL-2 were used to treat advanced colon cancer. The survival time of patients with immune response was significantly prolonged (26). A fifteen year retrospective analysis showed that the survival of metastatic colorectal cancer (mCRC) patients treated with the first-line GOLFIG regimen was significantly longer than those treated with the FOLFOX regimen without GM-CSF (27). Another long-term follow-up clinical research confirmed that the GOLFIG regimen was a reliable, underestimated treatment option in pre-treated mCRC patients. The study also demonstrated a significant anti-tumor activity in largely pre-treated patients, and both PFS and OS were positively correlated with baseline neutrophil counts and occurrence of immune-related adverse events (irAEs) (28). Sequential use of GM-CSF and IL-2 after chemotherapy could enhance the antigen-presenting function of dendritic cells, promote stronger and more effective immunological activity of antigen-specific CTL, and improve the positive development of tumor immunity and the reconstruction of immune function. The reduction of granulocytes and the peak of tumor immune reconstruction occur on the 7th to 14th day after chemotherapy, which is the best time to apply GM-CSF and IL-2 (26). Thus, in this study, we applied GM-CSF and IL-2 to patients who underwent individualized chemotherapy to enhance the treatment efficacy.

In other cancers, the cytokine environment as well as other biomarkers (platelet to lymphocytes ratio) seem to be of much concern in the prognosis of tumor disease which may also be applied to pancreatic cancer (29). In the preliminary basic experiment, Correale also found that lymphocytes, eosinophils and activated central memory T lymphocytes increased, while regulatory T cell subsets decreased during the decline of peripheral blood neutrophils, platelets and red blood cells. Patients with autoimmune responses during treatment had better therapeutic outcomes and longer survival time. Our study also found that some patients with better therapeutic effects had an increased proportion of eosinophils in peripheral blood after treatment. Patients with rash during treatment had better efficacy. The better treatment response in these patients may be related to the activation of non-specific immune response, which is worthy of further clinical discussion and application. Moreover, the immune factor GM-CSF could stimulate the production of granule-monocytes in peripheral blood and reduce the incidence of severe bone marrow suppression to ensure the safety and timeliness of treatment.

In conclusion, our retrospective study found that individualized selection of chemotherapy drugs combined with sequential immunotherapy based on BRCA1 mRNA expression level could control the disease and prolong survival of patients with unresectable pancreatic cancer. Our findings should be interpreted cautiously, because the number of patients we included was still small and could lead to biased and incomplete results. However, our study indicated that individualized chemotherapy combined with sequential immunotherapy based on BRCA1 gene expression level may be superior to unified adoption of AG regimen. The BRCA1 gene expression level is expected to be a biomarker for the decision-making of chemotherapy regimen in patients with unresectable pancreatic cancer. Further prospective studies with larger sample sizes are needed to confirm our findings.

## Conclusion

Our results suggest that individualized selection of chemotherapy combined with sequential immunotherapy according to BRCA1 mRNA expression level in the treatment of unresectable pancreatic cancer could control the disease and have controllable adverse reactions.

## Data availability statement

The raw data supporting the conclusions of this article will be made available by the authors, without undue reservation.

## Ethics statement

The studies involving human participants were reviewed and approved by the Ethics Committee of Nanjing Drum Hospital. The patients/participants provided their written informed consent to participate in this study.

## Author contributions

BL Research leader YQ Research leader, Disease diagnosis JD Research leader, Treatment intervention HS Treatment intervention, Follow up ZZ Treatment intervention, Follow up LianZ Treatment intervention, Follow up JS Treatment intervention, Follow up WK Treatment intervention, Follow up LY Treatment intervention QG Data collectiona LinZ Data collection, Statistical analysis, Paper writing. All authors contributed to the article and approved the submitted version.

## Conflict of interest

The authors declare that the research was conducted in the absence of any commercial or financial relationships that could be construed as a potential conflict of interest.

## Publisher’s note

All claims expressed in this article are solely those of the authors and do not necessarily represent those of their affiliated organizations, or those of the publisher, the editors and the reviewers. Any product that may be evaluated in this article, or claim that may be made by its manufacturer, is not guaranteed or endorsed by the publisher.
